# Whey protein consumption following fasted exercise reduces early postprandial glycaemia in centrally obese males: a randomised controlled trial

**DOI:** 10.1007/s00394-020-02304-2

**Published:** 2020-06-22

**Authors:** Dean M. Allerton, Daniel J. West, Emma J. Stevenson

**Affiliations:** 1 School of Biomedical, Nutritional and Sport Sciences, Newcastle University, Newcastle upon Tyne, UK; 2 Population Health Sciences Institute, Newcastle University, Newcastle upon Tyne, UK

**Keywords:** Whey protein, Postprandial, Exercise, Glycaemia, Insulin

## Abstract

**Purpose:**

Acute submaximal exercise and whey protein supplementation have been reported to improve postprandial metabolic and appetite responses to a subsequent meal independently. We aimed to examine the combination of these strategies on postprandial responses to a carbohydrate-rich breakfast.

**Methods:**

Twelve centrally obese males (age 41 ± 3 years, waist circumference 123.4 ± 2.9 cm), completed three trials in a single-blind, crossover design. Participants rested for 30 min (CON) or completed 30 min low–moderate-intensity treadmill walking (51 ± 1% ${{\dot{V}O}}_{\text{2peak}}$) followed immediately by ingestion of 20 g whey protein (EX + PRO) or placebo (EX). After 15 min, a standardised breakfast was consumed and blood, expired gas and subjective appetite were sampled postprandially. After 240 min, an ad libitum lunch meal was provided to assess energy intake.

**Results:**

During EX + PRO, post-breakfast peak blood glucose was reduced when compared with EX and CON (EX + PRO: 7.6 ± 0.4 vs EX: 8.4 ± 0.3; CON: 8.3 ± 0.3 mmol l^−1^, *p* ≤ 0.04). Early postprandial glucose AUC_0–60 min_ was significantly lower under EX + PRO than EX (*p* = 0.011), but not CON (*p* = 0.12). Over the full postprandial period, AUC_0–240 min_ during EX + PRO did not differ from other trials (*p* > 0.05). Peak plasma insulin concentrations and AUC_0–240 min_ were higher during EX + PRO than CON, but similar to EX. Plasma triglyceride concentrations, substrate oxidation and subjective appetite responses were similar across trials and ad libitum energy intake was not influenced by prior fasted exercise, nor its combination with whey protein supplementation (*p* > 0.05).

**Conclusion:**

Following fasted low–moderate-intensity exercise, consuming whey protein before breakfast may improve postprandial glucose excursions, without influencing appetite or subsequent energy intake, in centrally obese males.

**Trial registration number:**

NCT02714309.

## Introduction

It is well established that obesity is associated with dysregulation of a number of metabolic processes including glucose and lipoprotein metabolism, and thus increased risk of developing type 2 diabetes and cardiovascular disease [1]. Indeed, in developed countries, obesity is by far the most prevalent cause of insulin resistance [2], which is related to chronic, low-grade inflammation and macrophage infiltration in adipose tissue and increased circulating concentrations of pro-inflammatory cytokines [3, 4]. Central (abdominal) obesity is known to be particularly hazardous in the pathogenesis of insulin resistance and type 2 diabetes [5], with visceral fat tissue expressing higher levels of many cytokines [6] and a higher rate of lipolysis [7] than subcutaneous depots. The liver is, therefore, exposed to high levels of both non-esterified fatty acids and pro-inflammatory factors released from visceral fat, leading to elevated hepatic triglyceride and consequent deleterious effects on hepatic insulin sensitivity [8].

In the progressive decline from normal to impaired glucose tolerance that precedes the development of type 2 diabetes, it is postprandial rather than fasting glucose that appears to deteriorate first [9, 10]. Postprandial hyperglycaemia has been identified as an independent risk factor for cardiovascular disease in diabetic and non-diabetic populations [11–13], including when fasting glucose is in the normal range [14]. Excessive postprandial glucose excursions are, therefore, a strong predictor of future cardiovascular disease events [15]. Moreover, dysregulated lipoprotein metabolism is prevalent in those with central obesity and insulin resistance, with post-meal hypertriglyceridaemia increasingly recognised as a contributor to cardiovascular disease risk [16].

Exercise is a potent non-pharmacological strategy to reduce the burden of insulin resistance induced postprandial hyperglycaemia [17, 18] and lipidaemia [19]. Acute bouts of moderate-intensity exercise can upregulate a number of pathways that contribute to increased postprandial glucose disposal. This includes expression and translocation of GLUT4 to the cell membrane [20], which persists for several hours after cessation of exercise [21], in addition to some evidence of upregulated insulin signalling pathways following moderate-intensity exercise [22]. Additionally, exercise reduces postprandial lipaemia via increased hydrolysis of intramuscular triglyceride, due to increased lipoprotein lipase activity and reduced hepatic output of very low-density lipoproteins [23]. Regular training appears to impact postprandial glucose disposal via improvements in insulin sensitivity induced by adaptations including upregulation of muscle GLUT4 protein, increased enzyme capacities and muscle capillarization [20].

Nutritional strategies can also impact postprandial hyperglycaemia, and it has recently been demonstrated that prandial whey protein supplementation can significantly reduce subsequent glycaemic excursions in overweight men [24]. Whey protein contains amino acids and bioactive peptides which reduce postprandial glucose excursions via insulin-dependent and independent mechanisms [25]. Numerous studies have investigated the efficacy of whey protein supplementation on subsequent postprandial glycaemia in patients with type 2 diabetes [26–28], however, few trials have been conducted in centrally obese, non-diabetic individuals. Such individuals are at risk of being exposed to the adverse effects of postprandial hyperglycaemia [29]. Considering the effectiveness of both exercise and whey protein supplementation for improving postprandial glycaemia, a combination of these strategies may be a more effective approach.

The vast majority of studies investigating the effects of post-exercise whey protein supplementation have been conducted following resistance-type exercise, with beneficial effects on muscle protein synthesis [30] and lean mass maintenance [31, 32] previously described. However, the influence of whey protein supplementation following aerobic exercise on subsequent metabolic and appetite responses has received little attention. Reduced ad libitum energy intake has been observed 60 min after milk [33] or whey protein [34] consumption following prior moderate-intensity cycling exercise in recreationally active participants. Whether postprandial metabolic and appetite responses would be influenced by post-exercise whey protein consumption in habitually inactive obese individuals remains unclear. Given that a single bout of exercise may produce divergent responses in subsequent glucose tolerance [35], and that whey has been observed to influence postprandial glycaemia and insulinaemia consistently [36], the impact of post-exercise whey consumption on postprandial metabolic responses may be of significance.

Therefore, the aim of this study was to investigate the effect of fasted moderate-intensity exercise and subsequent whey protein supplementation on postprandial metabolic and appetite responses in centrally obese males.

## Materials and methods

### Participants

Male participants, aged 18–55 years, with central obesity and a low physical activity level were recruited. Central obesity was defined as waist circumference above the WHO threshold (102 cm) for abdominal obesity in males [37]. Physical activity level was assessed using the categorical scoring method following completion of the International Physical Activity Questionnaire [38]. Participants were excluded if they suffered from cardiovascular, metabolic or renal disorders, had a current illness, were taking medication that may affect metabolism or were a smoker. Additionally, participants were excluded if they self-reported regularly skipping breakfast (consuming breakfast on two or less occasions in the previous 7 days), having food allergies/intolerances or an eating disorder. All participants provided written informed consent prior to participation. Sample size was determined using a within-subject power analysis using a previous study in an overweight/obese population [39]. Following consumption of a whey protein preload with a mixed-macronutrient breakfast, a 16% reduction in glucose AUC was observed with a within-group variance of 11%. Statistical power was set at 80%, with a two-sided alpha level of 0.05.

## Experimental design

In a single-blind within-subject design, participants completed three trials, separated by at least 7 days. Participants were randomly assigned to pre-determined counterbalanced trial schedules that were created using an online randomisation tool for researchers (https://www.randomization.com/). Two exercise trials involved a 30 min bout of brisk treadmill walking followed by consumption of a whey protein (EX + PRO) or placebo (EX) preload beverage. A third trial involved participants remaining sedentary for the same duration followed by consumption of the placebo beverage (CON). A standardised, carbohydrate-rich, breakfast meal was provided 15 min after consumption of test preloads (whey protein or placebo) under all conditions. Participants remained sedentary for a further 240 min, followed by consumption of an ad libitum mixed-macronutrient lunch meal. This research took place within the Faculty of Health and Life Sciences research laboratories, Northumbria University, UK, with recruitment, data collection and analysis taking place between March and August 2016.

## Pre-trial procedures

Prior to experimental visits, participants completed a submaximal treadmill walking test to determine their prescribed walking speed for the main trials. Four steady-state walking stages were completed on a motorised laboratory treadmill (Pulsar 3p, h/p/cosmos, Germany). Participants began walking at 3–4 km h^−1^ with walking speed increased by 0.5 or 1 km h^−1^ at the end of each 3 min stage according to the discretion of the researcher, and based on the rating of perceived exertion (RPE) in the final 30 s of each stage [40]. Throughout the test, expired gas was sampled using a breath by breath gas analyser (Oxycon Pro, CareFusion, USA) and heart rate was recorded using short-range telemetry (Polar RS400, Polar Electro, Finland). The relationship between oxygen consumption and heart rate was extrapolated to age-predicted maximum heart rate to estimate ${{\dot{V}O}}_{\text{2peak}}$ for each participant. The walking speed eliciting an intensity of 50% ${{\dot{V}O}}_{\text{2peak}}$ was determined from the relationship between oxygen consumption and walking speed, and selected as the prescribed walking speed during main trials.

Participants were instructed to avoid strenuous physical activity in addition to caffeine and alcohol consumption for 24 h prior to each laboratory visit. To standardise pre-trial nutritional intake, an identical mixed-macronutrient evening meal was provided before each visit, and participants were asked to consume this 12 h prior to arrival. This consisted of a beef lasagne meal (Tesco, UK) with a honey-flavoured oat bar (Nature Valley, USA), providing 3501 kJ energy (837 kcal; 37% carbohydrate, 19% protein, 44% fat).

### Main trial procedures

Participants arrived at the laboratory following an overnight fast, where a cannula was inserted into an antecubital vein. Baseline venous and capillary blood samples were taken, and measures of subjective appetite and expired gas were collected. During CON participants remained rested, whilst during EX + PRO and EX, a 30 min bout of steady-state brisk treadmill walking at 50% ${{\dot{V}O}}_{\text{2peak}}$ was performed. The mode, intensity and duration of exercise was designed to be achievable for habitually sedentary individuals, while also conforming to UK recommendations for prescribed levels of daily physical activity for prevention of obesity [41].

**Fig. 1:**
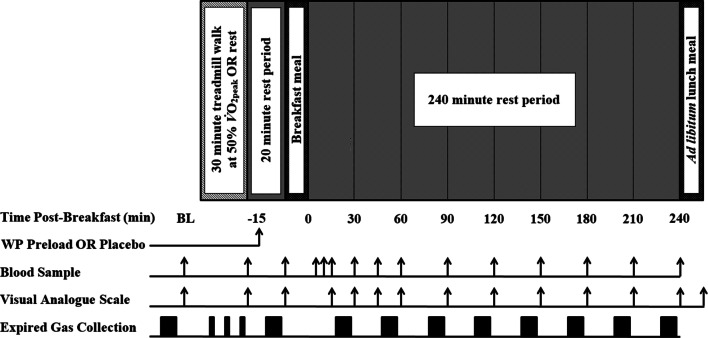
Schematic representation of experimental trials. *WP* whey protein, *BL* baseline

Within 5 min of exercise completion, participants consumed a test beverage containing whey protein during EX + PRO, and a placebo beverage during EX. During CON, a placebo beverage was consumed at the corresponding time point. The remainder of the trial procedure was identical under all conditions. A standardised breakfast was consumed 15 min after test beverage ingestion, and participants subsequently remained seated and rested for 240 min with blood, expired gas and VAS sampled at regular intervals (Fig. 1). After 240 min, an ad libitum lunch meal was provided to assess energy intake.

### Test meals

In the EX + PRO trial, the test beverage consisted of 20 g whey protein isolate (Lacprodan SP-9225 Instant; Arla Food Ingredients Group, Viby, Denmark) combined with 150 ml water and 0.5 ml energy-free strawberry flavouring (FlavDrops, Myprotein, UK). In the EX and CON trials, an isovolumetric bolus of similarly flavoured water was consumed as a placebo. All test drinks were provided in opaque bottles. An additional 200 ml drinking water was administered after each test beverage to eliminate any after taste.

A standardised portion of rolled porridge oats with semi-skimmed milk and honey was provided as breakfast under all conditions, providing 1958 kJ of energy (468 kcal; 70% carbohydrate, 17% fat, 13% protein). Participants were encouraged to consume this meal within 10 min, and 250 ml drinking water was provided alongside the porridge. A timer was started upon completion of this meal.

A homogenous pasta meal was provided ad libitum at lunch to assess energy intake. This consisted of cooked dried pasta, a tomato-based sauce, cheddar cheese and olive oil (Tesco, UK) as described previously [42], providing 53%, 14% and 33% energy from carbohydrate, protein and fat, respectively. Participants were initially provided with a 400 g (2845 kJ, 680 kcal) portion of the pasta and were instructed to eat until they felt ‘comfortably full’ on each occasion. The serving bowl was topped up with fresh pasta prior to completion, thus removing the effect of bowl clearance as a stimulus for food intake termination. All portions of cooked pasta (served or unserved) were weighed immediately before and after consumption to determine energy intake.

### Indirect calorimetry

Expired gas was sampled at regular intervals throughout resting and exercise periods of the protocol (Fig. 1) using an online gas analyser (Oxycon Pro, CareFusion, USA). Samples were collected at baseline following 10 min rest, immediately after the test beverage, 20 min post-breakfast, and every subsequent 30 min. Resting expired gas was sampled for 10 min periods, with data from the first and last minute of each period discarded. During treadmill walking, expired gas was collected for 5 min periods at 5, 15 and 25 min during the exercise bout, with the first and last minutes discarded. Resting substrate oxidation rates were calculated as per the equations of Frayn [43] and exercising substrate oxidation rates were calculated as per Jeukendrup and Wallis [44], accounting for the increased glycogen contribution to carbohydrate metabolism during moderate-intensity exercise.

### Blood sampling and analysis

A cannula (Vasofix 22G, B.Braun Melsungen AG, Germany) was inserted into a vein in the antecubital fossa upon participant arrival. At regular intervals (Fig. 1), 10 ml of whole venous blood was transferred into EDTA-coated tubes (Vacutainer, Becton Dickinson, USA) and immediately centrifuged for 10 min at 1734* g* and 4 °C (Allegra X-22R, Beckman Coulter, USA). Plasma was stored at − 80 °C for subsequent analysis. Fingertip capillary blood was sampled (20 µl) at corresponding time points with blood glucose concentration immediately determined (Biosen C_line analyser, EKF Diagnostics, UK). Additional samples were collected at 5 and 10 min post-meal to increase the resolution of the blood glucose curve. A commercially available ELISA (IBL International, Hamburg, Germany) was used to determine venous plasma insulin concentrations, with intra- and inter-assay variation (CV) of 7.8% and 8.8%, respectively. Enzymatic colorimetric assays were performed on an automated analyser (RX Daytona, Randox Laboratories, UK) to determine plasma glycerol and triglyceride concentrations.

## Subjective appetite

Subjective appetite ratings were assessed using VAS, with a combined appetite score subsequently calculated as described previously [45]. Ratings for hunger, fullness, PFC and satisfaction were collected at corresponding time points to venous blood samples (Fig. 1), with a final VAS completed following termination of the lunch meal.

### Statistical analysis

Total AUC was calculated from blood analyte and subjective appetite data for the early (0–60 min), intermediate (0–120 min) and full (0–240 min) postprandial periods using the trapezoidal method [46]. Fasting and postprandial concentrations of plasma glucose and insulin were used to calculate the Matsuda Insulin Sensitivity Index (ISI) [47]. Missing data (2.5% of all planned observations) were imputed using the linear interpolation technique. Completer only statistical analysis was performed using SPSS (version 21, IBM, USA). Glucose, insulin, triglyceride, glycerol and subjective appetite responses were analysed using two-way repeated measures analysis of variance (ANOVA) with condition and time as factors. Baseline comparisons between trials, AUC for all variables, measures of energy balance and substrate metabolism were assessed using one-way repeated measures ANOVA. Post hoc comparisons were conducted upon identification of significant main effects and were adjusted for multiple comparisons using the Bonferroni correction. The level of statistical significance was set at *p* < 0.05 and data are presented as mean ± standard error of the mean (SEM).

## Results

### Participant characteristics

In total, 15 participants were recruited to take part in the study. Three participants did not complete the protocol, with one dropping out prior to, and two following the pre-trial submaximal walking test. Participant characteristics for all participants who completed the study (*n* = 12) are displayed in Table 1.

**Table 1:** Participant characteristics

	All participants (*n* = 12**)**
Characteristics	
Age (years)	41 ± 3
Body mass (kg)	121.9 ± 3.2
Stature (cm)	179.5 ± 1.7
BMI (kg m^−2^)	37.8 ± 0.6
Waist circumference (cm)	123.4 ± 2.9
Waist/hip ratio	1.01 ± 0.02
${{\dot{V}O}}_{\text{2peak}}$ (ml kg^−1^ min^−1^)	25.5 ± 1.1
Fasting variables^a^	
Blood glucose (mmol l^−1^)	5.2 ± 0.2
Plasma insulin (pmol l^−1^)	122.6 ± 16.4
HOMA-IR	4.7 ± 0.7
Plasma triglyceride (mmol l^−1^)	1.50 ± 0.18

^a^Fasting values are presented as mean of fasting samples for each main trial

### Blood glucose and plasma insulin

**Fig. 2:**
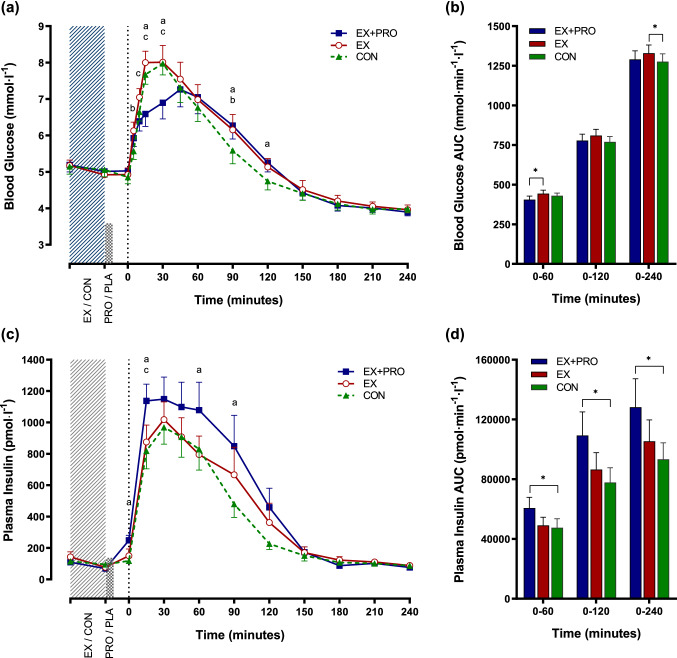
Mean ± SEM (*n* = 12) temporal changes in blood glucose (**a**) and plasma insulin (**c**) concentrations, with associated AUC for glucose (**b**) and insulin (**d**). Significant differences (*p* < 0.05) between conditions at individual time points are defined as follows; **a** EX + PRO vs CON; **b** EX vs CON; **c** EX + PRO vs EX. Significant differences between bars are denoted with an asterisk. Dotted line indicates time of breakfast consumption. *EX + PRO* exercise with whey protein preload trial, *EX* exercise trial, *CON* resting trial

Glucose displayed a significant condition × time interaction effect (*p* < 0.001), time effect (*p* < 0.001) and main effect of condition (*p* = 0.009; Fig. 2a). The post-breakfast increase in glucose was reduced in EX + PRO compared with placebo trials at 15–30 min post-breakfast, and a significantly reduced peak was observed in this condition (EX + PRO: 7.6 ± 0.4 vs EX: 8.4 ± 0.3, CON: 8.3 ± 0.3 mmol l^−1^, *p* ≤ 0.04). Early postprandial glucose AUC_0–60 min_ was significantly lower under EX + PRO when compared to EX (Fig. 2b; *p* = 0.011), but not significantly different from CON (*p* = 0.12) after correcting for multiple comparisons. Glucose was significantly lower during CON than EX + PRO and EX at 90 min, and lower than EX + PRO at 120 min post-breakfast (all *p* < 0.05). Values declined significantly below baseline levels after 180 min in all conditions. Over the full postprandial period (0–240 min), glycaemia was greater during EX compared with CON (*p* = 0.002) but not significantly higher than EX + PRO (Fig. 2b; *p* = 0.241).

Insulin displayed a significant interaction of condition and time (*p* = 0.006), and main effects for time (*p* < 0.001) and condition (*p* = 0.027; Fig. 2c). Plasma insulin concentrations were not different immediately post-exercise, but were significantly greater immediately prior to breakfast in EX + PRO compared with CON (EX + PRO: 249 ± 32 vs CON: 118 ± 13 pmol l^−1^, *p* < 0.001), but not EX (151 ± 44 pmol l^−1^, *p* = 0.379). A larger peak in insulin was observed during EX + PRO compared with CON (EX + PRO: 1374 ± 602 vs CON: 1050 ± 420 pmol l^−1^, *p* = 0.004) and insulin AUC was greater during EX + PRO than CON, but not EX, during the acute (0–60 min), intermediate (0–120 min) and full (0–240 min) postprandial analyses (Fig. 2d). There were no differences observed between conditions in whole-body insulin sensitivity following breakfast consumption (Matsuda-ISI: EX + PRO: 2.3 ± 0.3, EX: 2.3 ± 0.3, CON: 2.6 ± 0.4; *p* = 0.344).

### Plasma triglyceride and glycerol

**Fig. 3:**
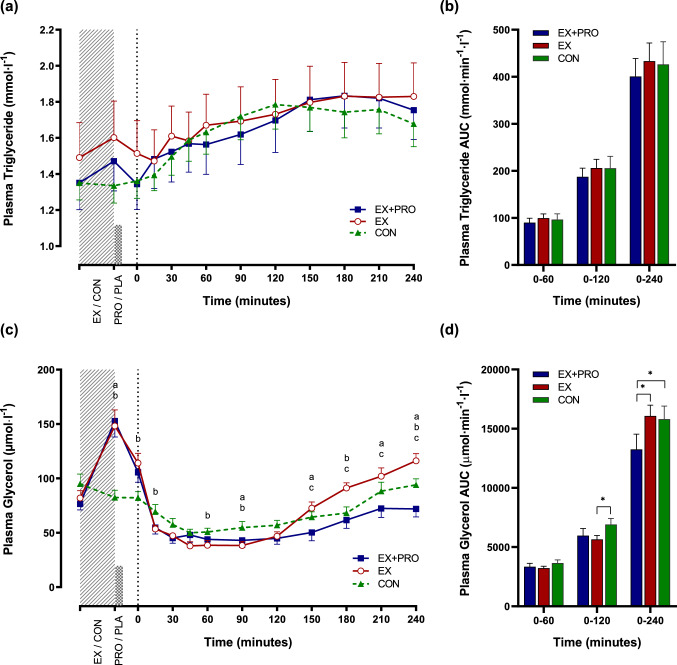
Mean ± SEM (*n* = 12) temporal changes in plasma triglyceride (**a**) and glycerol (**c**) concentrations with associated AUC for triglyceride (**b**) and glycerol (**d**). Significant differences (*p* < 0.05) between conditions at individual time points are defined as follows; **a** EX + PRO vs CON; **b** EX vs CON; **c** EX + PRO vs EX. Significant differences between bars are denoted with an asterisk. Dotted line indicates time of breakfast consumption. *EX + PRO* exercise with whey protein preload trial, *EX* exercise trial, *CON* resting trial

There was no effect of condition or condition x time interaction effect on plasma triglyceride responses (Fig. 3a). There were no differences between conditions at baseline, immediately post-exercise, or immediately prior to breakfast in plasma triglyceride concentrations (*p* > 0.05). Following breakfast, responses were significantly affected by time (*p* < 0.001), such that triglyceride was significantly increased above baseline at 120–210 min post-breakfast in all conditions (Fig. 3a; all *p* < 0.05). AUC was similar across conditions (Fig. 3b; all *p* > 0.05).

Post-breakfast glycerol concentrations were influenced by condition (*p* = 0.004) and time (*p* < 0.001), with a condition x time interaction effect also observed (*p* < 0.001; Fig. 3c). Circulating glycerol concentrations did not differ between conditions at baseline, but were significantly greater in exercise trials than CON immediately post-exercise (Fig. 3b; both *p* ≤ 0.004), remaining elevated immediately prior to breakfast in EX (*p* = 0.019). AUC_0–240 min_ was lower in EX + PRO compared to both EX (*p* = 0.023) and CON (*p* = 0.005; Fig. 3d).

### Energy balance and substrate oxidation

The amount of energy expended did not differ between EX + PRO and EX throughout (*p* > 0.05), but was greater during the exercise period in EX + PRO and EX than CON, fully accounting for the significantly greater total energy expenditure in exercise trials compared with CON (both *p* < 0.001; Table 2). No differences were detected between conditions in absolute energy intake at the ad libitum lunch meal (*p* = 0.886), signifying that participants did not compensate for the excess energy expended in exercise trials at the subsequent lunch meal. When total intake over the whole trial (breakfast, test drink and lunch) was compared, no between-condition differences were present (*p* = 0.491).

**Table 2:** Energy intake, expenditure and substrate metabolism during the exercise period (30 min), post-breakfast period (240 min) or full protocol (~ 300 min)

	EX + PRO	EX	CON
Energy intake (kJ)			
Breakfast	2302 ± 0	1958 ± 0	1958 ± 0
Lunch	4623 ± 356	4728 ± 385	4569 ± 343
Total	6925 ± 356	6686 ± 385	6527 ± 343
Energy expenditure (kJ)			
Exercise period	938 ± 48^a^	914 ± 43^b^	203 ± 14^a,b^
Post-breakfast	1657 ± 87	1695 ± 83	1572 ± 73
Total	2690 ± 134^a^	2712 ± 122^b^	1866 ± 86^a,b^
Carbohydrate oxidation (g)			
Exercise period	31.4 ± 2.6^a^	30.1 ± 2.7^b^	6.1 ± 1.1^a,b^
Post-breakfast	55.4 ± 4.3	56.2 ± 5.7	57.1 ± 4.3
Total	89.3 ± 6.9^a^	89.0 ± 8.2^b^	65.5 ± 5.5^a,b^
Fat oxidation (g)			
Exercise period	10.7 ± 1.3^a^	10.7 ± 1.3^b^	2.9 ± 0.4^a,b^
Post-breakfast	21.0 ± 2.0	21.7 ± 2.1	18.0 ± 2.1
Total	33.1 ± 3.3^a^	33.9 ± 3.5^b^	22.4 ± 2.6^a,b^

Data presented as mean ± SEM. Matching superscript letters within a row denotes significant difference between conditions (*p* < 0.05)

*EX + PRO* exercise with whey protein preload trial, *EX* exercise trial, *CON* resting trial

Rates of fat and carbohydrate oxidation did not differ between conditions at baseline (*p* = 0.593 and *p* = 0.879, respectively). Greater amounts of fat and carbohydrate were utilised over the course of each exercise trial in comparison to resting control (all *p* < 0.05), however, substrate metabolism was not influenced by consumption of whey protein, with similar fat and carbohydrate oxidation observed between EX + PRO and EX throughout (all *p* > 0.05; Table 2).

### Subjective appetite ratings

**Fig. 4:**
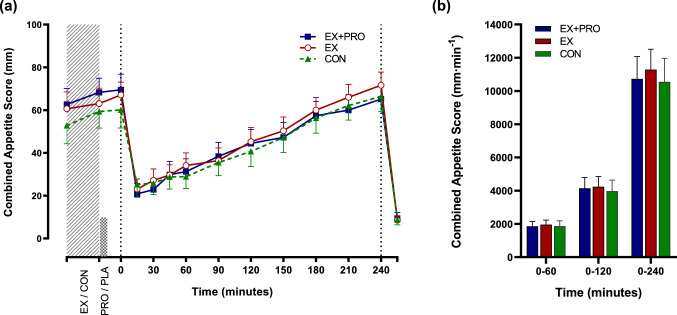
Mean ± SEM (*n* = 12) temporal changes (**a**) and AUC (**b**) for combined appetite score. Dotted lines indicate time of meal consumption. *EX + PRO* exercise with whey protein preload trial, *EX* exercise trial, *CON* resting trial

A significant effect of time on combined appetite responses was observed (*p* < 0.001). Appetite decreased similarly following breakfast in all trials, returning to baseline levels at 90–240 min post-breakfast, before decreasing similarly after the ad libitum lunch meal (Fig. 4a). There was no difference in AUC for combined appetite score (Fig. 4b) or individual components of subjective appetite under all conditions (all *p* > 0.05).

## Discussion

The main finding observed was that fasted exercise followed by pre-breakfast whey protein supplementation improved early postprandial glycaemia. This was illustrated by a reduction in the post-breakfast glucose peak in comparison to both placebo trials, and lower glucose AUC_(0–60 min)_ when compared to exercise alone. In addition, it is shown that fasted exercise in conjunction with post-exercise whey protein supplementation does not influence subsequent energy intake in centrally obese males.

An acute bout of exercise moderately impaired glucose tolerance, indicated by a higher glucose AUC_0–240 min_ in the exercise control compared to resting control, while insulin AUC remained unchanged. Glucose peak and early AUC were not different between these conditions, indicating that this effect was manifested by a more prolonged elevation in blood glucose after prior exercise. When whey protein was ingested following exercise, this impairment appears to be negated, with glycaemia not differing from control overall. Additionally, glycaemia was ~ 9% lower in the early (0–60 min) period following breakfast when compared to exercise without additional protein, whilst peak postprandial glucose was significantly reduced compared to other conditions.

The increased glycaemic response immediately following a bout of exercise is in accordance with previous findings in healthy trained [48] and obese normoglycaemic males [49]. Rose et al. [48] observed a 30% elevation in glucose appearance during an OGTT following 30 min of cycling exercise, albeit at a higher intensity (70% ${{\dot{V}O}}_{\text{2peak}}$) than the current study. The counterregulatory response to sustained exercise (more than ~ 20 min) involves increases in glucagon, catecholamines, and cortisol secretion, among other hormones [50], which may result in a rate of glucose appearance [51] which exceeds disappearance [52]. Exercise-induced elevation in catecholamine levels has also been shown to enhance the appearance of orally ingested glucose in an animal model via stimulation of sodium-glucose linked transporter type 1 (SGLT1) [53], whilst increased postprandial splanchnic perfusion after exercise may also explain increased glucose absorption [54]. It is currently unclear how long this effect persists for, however, it is likely to be transient in nature [49], and blood glucose levels were similar between exercise and control trials beyond 120 min post-breakfast in the current study. Moreover, should obese individuals take part in regular exercise training, then improvements in aerobic fitness and potential weight loss are likely to have independent effects on improving glucose metabolism [20, 55]. Nevertheless, knowledge of the acute effect of exercise on glucose tolerance when food is ingested immediately or shortly after exercise, is of significance when aiming to ameliorate the adverse effects of postprandial hyperglycaemia on metabolic health [56, 57].

The observed reduction in acute glycaemia following post-exercise whey protein supplementation could be attributed to a combination of mechanisms, including the direct effects of amino acids, particularly leucine, on β-cell stimulation [58] and activation of the incretin response [59]. Circulating insulin was increased 15 min after consumption of the whey preload, coinciding with the timing of breakfast consumption. The post-breakfast rise in insulin secretion also occurred earlier in the EX + PRO trial, with higher concentrations observed at 15 min post-breakfast compared with both non-protein conditions. The post-breakfast elevation in EX + PRO persists for up to 90 min, without further reductions in postprandial glycaemia. Increased postprandial insulinaemia in the absence of a reduction in glycaemia is suggestive of compromised insulin sensitivity, an effect that has previously been observed following acute whey protein ingestion [60]. Thus, there is potential for the chronic exposure to the insulinotrophic effects of whey protein to have a desensitising effect on sites of insulin secretion or action, however, the implementation of longer-term supplementation protocols are required to investigate this potential effect. As the acute glucose response was attenuated in EX + PRO compared with EX, in the context of a potentially increased rate of post-exercise glucose absorption, this is suggestive of delayed gastrointestinal transport of orally ingested glucose. The rate of gastric emptying exerts considerable influence on the magnitude of postprandial glucose excursions [61, 62], accounting for ~ 35% of variance in glycaemic response to carbohydrate-containing meals in healthy individuals [63].

Large fluctuations in circulating glucose concentrations have deleterious effects on endothelial function and oxidative stress in both healthy individuals and T2DM patients [57]. The attenuation of peak glucose excursion compared to both exercise and resting control conditions in the current study may, therefore, indicate a role for pre-meal supplementation of whey protein both at rest, and when consuming meals shortly after bouts of low–moderate-intensity exercise, in obese individuals. The stimulus was not large enough to detect a reduction in glycaemia over the full postprandial period, however, it was sufficient to negate the increase observed in post-exercise glycaemia without additional whey protein.

There was no effect of whey protein supplementation on postprandial triglyceride in the current study. This reflects previous findings when similar doses of whey protein have been administered prior to the same breakfast meal [24] or meals with considerably higher fat loads [64–66], albeit without prior exercise. Conversely, studies administering considerably higher doses of whey protein (45 g) alongside high fat loads (80 g) have shown significant reductions in postprandial lipaemia [67, 68]. The present study was designed to reflect practical application, thus the amount of whey protein and the composition of test meals were realistic in the context of habitual eating habits [69].

Elevated glycerol concentrations immediately post-exercise are indicative of increased lipolysis and lipid substrate availability for exercise, confirmed by the observed increase in fat oxidation during exercise. Increased fat oxidation is associated with the postprandial triglyceride-lowering effects of exercise [70], however, postprandial substrate utilisation was not significantly different between exercise and control trials in the present study, and triglyceride responses were similar between conditions. A large body of evidence implicates exercise in the attenuation of postprandial lipaemia [19, 23], however, the clear majority of studies have administered a test meal > 4 h after cessation of exercise. Those studies that have shown a more acute effect have administered meals considerably greater in fat content (> 95 g) and prescribed a greater workload during aerobic exercise bouts than the current study [71, 72]. Furthermore, replacement of the exercise-induced energy deficit via mixed-macronutrient [73] or carbohydrate [74] feeding attenuates or abolishes improvements in postprandial triglyceridaemia. In the current study, participants consumed breakfast containing more than double the amount of energy expended during treadmill walking, which may account for the similar lipaemic responses in exercise and resting conditions. It has also been observed that consumption of a meal immediately after exercise diminishes the shift from carbohydrate to fat oxidation that usually follows exercise [75], which may explain the lack of significant differences in substrate utilisation in resting and exercise conditions in this study. Although it could be speculated that the intensity of the exercise bout was not high enough to have a prolonged metabolic effect, it has previously been established that total energy expenditure rather than intensity is of primary importance to the triglyceride-lowering effect of prior exercise [19].

Appetite was unaffected by prior exercise in the current study, which appears to be consistent with previous evidence suggesting that appetite is not altered by acute moderate-intensity exercise [76]. In accordance with the comparable subjective appetite responses, lunch meal energy intake 4 h post-breakfast was similar between conditions. The fact that participants did not compensate for the deficit created by prior exercise reflects the findings of the majority of exercise studies [77] and brisk walking protocols [78] in lean, healthy individuals, whilst limited evidence exists to support a similar tendency in obese individuals. The moderate energy deficit created by the exercise bout in the present study, in addition to the short duration between exercise and consumption of a standardised breakfast, is likely to have influenced this response. It may also be a possibility that energy expenditure from exercise is gradually compensated for over several meals or even days, however such compensation is likely to only partially account for energy expended [79]. Nevertheless, the difference in energy balance in the exercise control trial compared with resting control was equivalent to the net amount expended during exercise, highlighting the potential efficacy of brisk walking to create acute energy deficits in obese individuals.

A limitation of the current study is the lack of a non-exercise whey protein condition, which makes uncoupling of the effects of whey protein and prior exercise on glycaemia problematic. Whether the blood glucose response would be further reduced by consuming a whey protein preload in the absence of prior exercise can only be speculated, however, we have previously observed reductions in peak glucose and postprandial glycaemia at rest following whey protein consumption prior to the same breakfast meal in centrally obese males [24]. Furthermore, implementation of a longer investigation period may have been warranted, as the transient effects of a single bout of exercise on insulin sensitivity may last for up to 72 h [21], indicating that these beneficial effects may occur beyond the time frame examined here. The timing of post-exercise feeding may have limited the ability to identify significant effects of exercise on postprandial lipaemia and subsequent intake, however, the consumption of a meal or snack following fasted morning exercise is likely, ensuring that these findings hold relevance in a free-living setting. The fact that the study was ran in a single-blind fashion may also be considered a limitation, however, efforts were made to eliminate sources of bias including analysing all samples from a participant on a single run where possible, and employing an additional (blinded) researcher to independently measure appetite responses to verify the measurements made by the primary researcher.

The use of paired *t* tests to conduct pairwise comparisons for differences within main effects may be considered a limitation due to the assumption that systematic differences in responses between testing visits are absent. This was mitigated as far as possible by including a considerable wash out period between visits of 7 days, in addition to counterbalancing trial order regimens. Additionally, the study was powered to detect differences in the primary outcome of postprandial glycaemia, with the consequent likelihood that secondary outcomes may have been underpowered to detect differences.

The ecological validity of these findings is reinforced by utilising a dose of protein that could realistically be supplemented prior to a meal, along with an exercise load that is realistic and tolerable in the population of interest. Care was also taken to use foods that were typical of those consumed at breakfast and lunch meals across the population. Whilst the effects of high levels of protein intake on health is of interest, current evidence suggests that whey protein supplementation and higher total protein is not detrimental to bone health [80]. Acute trials such as the current study provide valuable information regarding the effects of whey protein consumption on immediate post-meal responses, however, consideration should be given to the fact that prevention of deteriorating metabolic health may require chronic improvements in postprandial glycaemia and other markers, which cannot be observed in the acute laboratory setting. Studies assessing the longer term effects of whey supplementation are relatively few in number, with only a small number in overweight/obese [81–83] or diabetic [84] individuals. Although some inconsistencies are apparent, the limited evidence to date appears to show that chronic supplementation of the diet with whey protein is associated with metabolic health benefits including improved fasting lipid profile and insulin sensitivity, with possible effects on food intake and body mass. There is increasing focus on non-pharmaceutical methods and functional foods in this area, and such work may be advanced through development of an optimal chronic supplementation strategy, while development of food products incorporating whey protein may enhance adherence to supplementation.

In summary, an isolated bout of brisk walking exercise moderately impaired post-exercise glucose tolerance, however a whey protein preload consumed immediately post-exercise negates this effect. Furthermore, acute postprandial glycaemia was attenuated following whey protein consumption, whilst energy intake at a later lunch meal was not influenced by the intervention.
